# Estimating income-related and area-based inequalities in mental health among nationally representative adolescents in Australia: The concentration index approach

**DOI:** 10.1371/journal.pone.0257573

**Published:** 2021-09-21

**Authors:** Md Irteja Islam, Gail M. Ormsby, Enamul Kabir, Rasheda Khanam

**Affiliations:** 1 Maternal and Child Health Division (MCHD), International Centre for Diarrhoeal Disease Research, Bangladesh (icddr,b), Mohakhali, Dhaka, Bangladesh; 2 Centre for Health Research and School of Business, University of Southern Queensland, Toowoomba, Queensland, Australia; 3 Professional Studies, School of Education, Faculty of Business, Education and Law, University of Southern Queensland, Toowoomba, Queensland, Australia; 4 School of Sciences, University of Southern Queensland, Toowoomba, Queensland, Australia; The University of Sydney, AUSTRALIA

## Abstract

Despite the awareness of the importance of mental health problems among adolescents in developed countries like Australia, inequality has not been widely researched. This study, is therefore, aimed to measure and compare household income-related and area-based socioeconomic inequalities in mental health problems (bullying victimization, mental disorders–single and multiple, self-harm and suicidality–ideation, plan and attempt) among Australian adolescents aged 12–17 years. Young Minds Matter (YMM)—the 2^nd^ national cross-sectional mental health and well-being survey involving Australian children and adolescents conducted in 2013–14, was used in this study to select data for adolescents aged 12–17 years (n = 2521). Outcome variables included: bullying, mental disorders, self-harm, and suicidal ideation, plan and attempt. The Erreygers’s corrected concentration index (CI) approach was used to measure the socioeconomic inequalities in mental health problems using two separate rank variables–equivalised household income quintiles and area-based Index of Relative Socioeconomic Advantage and Disadvantage (IRSAD) quintiles. The prevalence of mental health problems in the previous 12-months among these study participants were: bullying victimization (31.1%, 95% CI: 29%-33%), mental disorder (22.9%, 95% CI: 21%-24%), self-harm (9.1%, 95% CI: 8%-10%), suicidal ideation (8.5%, 95% CI: 7%-10%), suicidal plan (5.9%, 95% CI: 5%-7%) and suicidal attempt (2.8%, 95% CI: 2%-3%). The concentration indices (CIs) were statistically significant for bullying victimization (CI = -0.049, p = 0.020), multiple mental disorders (CI = -0.088, p = <0.001), suicidal ideation (CI = -0.023, p = 0.047) and suicidal attempt (CI = -0.021, p = 0.002), implying pro-poor socioeconomic inequalities based on equivalized household income quintiles. Similar findings revealed when adolescents mental health inequalities calculated on the basis of area based IRSAD (Index of Relative Socio-economic Advantage and Disadvantage) quintiles. Overall, adolescents from economically worse-off families experienced more mental health-related problems compared to those from economically better-off families. This has implications for prevention strategies and government policy in order to promote mental health and provide equitable healthcare facility.

## Introduction

Globally, socioeconomic inequality has become one of the most widely debated topics in social sciences, public health research and has broad implications for policy formation [1–3]. Low socioeconomic factors have widespread repercussions not only of adults but on adolescent’s education outcomes, health, and wellbeing [4].

An analysis of socioeconomic inequalities of adolescent health across 34 high-income countries between 2002–2010, showed that mental and physical health issues increased during this period [5, 6]. Additionally, it was noted that larger differences in socioeconomic status (SES) were associated with impaired physical activity and psychological disorders [6]. Reiss’s review of 52 papers, demonstrated the linkage of SES and adolescent’s mental health problems but also highlighted the need for further in-depth analysis of the socio-determinants of mental health [2]. As inequality worsens, it is likely that adolescents’ psychological and physical symptoms worsen [7]. A limited understanding of the determinants of mental health research among adolescents makes it difficult to plan appropriate public health interventions [3].

Limited studies have explored the severity of socioeconomic inequalities on adolescents’ health and wellbeing. However, inequalities have been observed in both developed and developing countries [8, 9]. Ongoing research has shown associations between income and depression or suicidality (ideation, plan and attempt), and delinquency as well as internally and externally directed violence during childhood and adolescence including traditional bullying and cyberbullying [10–12]. Worldwide, suicide is the second most common cause of death among young people resulting in a large human cost and lost productivity [3, 13]. Increasingly, research supports the notion that socioeconomic issues during adolescence impact not only development but also predicts future adversities including mental health disorders [14, 15], self-directed harm [16, 17] including suicidality [10], delinquency [11] and externalised violence in the form of bullying victimization [10]. A Netherlands study [18] found that parental socioeconomic status, adolescent subjective SES, and adolescent educational level were important indicators of inequalities in adolescent mental health. A Canadian study examined the association between cyberbullying, school bullying with suicidal ideation among middle and high school students, finding significant links [19]. Other studies have found similar links between school bullying and suicidality including suicide [20, 21].

Worldwide, mental health disorders account for a considerable percentage of the global burden of disease (mental, neurological and substance use disorders accounting for 13%, depression accounting for 4.3% of the global burden in 2004) [3]. In 2010, the global direct and indirect economic cost of mental disorders were estimated to be US$2.5 trillion and are expected to double by 2030 [1], illustrating the need for better prevention measures. In 2013, the World Health Organization launched the Mental Health Action Plan 2013–2020 to address the socio-determinants of mental health that impact the individual’s overall health and wellbeing as the treatment gap for neurologic, mental and substance used disorders were found to be higher compared to other health issues [3, 22]. The need for evidence-based research was highlighted, to inform universal health delivery strategies and appropriate community-based interventions [3]. Evidenced-based policy measures are needed to tackle the underlying causes of inequality among households/population groups to improve socioeconomic mobility of adolescents into adult life [10].

In Australia, the 1997 National Survey of Mental Health and Wellbeing was conducted among individuals aged 18 years of over and brought great awareness of mental health disorders [23]. Moreover, the 2013–2014 Australian Child and Adolescent Survey of Mental Health and Wellbeing surveyed a sample of 5500 children and adolescents aged 4–17 years highlighted child and adolescent mental health issues (bullying, mental disorder, self-harm and suicidality–ideation, plan and attempt) as a significant public health problem [24]. Additionally, a recent paper estimated the prevalence of major depressive disorder (11.5%), ADHD (6.5%), anxiety disorder (7.1%), conduct disorder (1.9%), suicidality (8%), and non-suicidal self-harm (7.8%) in adolescents aged 12–17 years [25], and was found to be like the research demonstrating the need for better public health interventions [26, 27]. However, regarding mental health inequalities in Australia, there are limited insightful and beneficial records are available. To be more precise, most descriptive studies involving adults have shown that certain classes, such as the aged, the unemployed, the divorced, people with reduced education and living remotely have higher rates of mental and personality disorders in Australia [28–30]. While to date, to the best of our knowledge, very limited number of studies has been thoroughly investigated the extent of such mental health differences among adolescents in Australia [31, 32]. Therefore, this study aimed to estimate and compare income -related and area-based socioeconomic inequalities in mental health problems in terms of bullying victimization, mental disorders, self-harm, suicidality (ideation, plan and attempt) among Australian adolescents aged 12–17 years using a nationally representative sample.

## Methods

### Data source and study participants

The Young Minds Matter (YMM) is the nationally representative household-based cross-sectional children and adolescents survey of mental health and well-being in Australia. The YMM conducted in 2013–14 in collaboration with Telethon Kids Institute, University of Western Australia (UWA), Roy Morgan Research, and the Australian Government Department of Health (AGDH).

In summary, the YMM implemented the multi-stage, random sampling technique for Australian households with children and adolescents aged between 4-17-year-olds. In the household, the sample included a single child/adolescent randomly selected when there was more than one qualified sample [24, 33]. A standardized questionnaire was completed by a face-to-face interview with 6310 parents (55% of eligible households) of 4-17-year-olds. In addition, a computer-based self-reported questionnaire has been privately completed by 2967 adolescents (89% of eligible households) aged between 11-17-years. The survey excluded homeless children/adolescents, children/adolescents from the distant places and residents of all households or organizations who cannot be interviewed in English. All the respondents (parents and self-reported adolescents) participated voluntarily in the survey, where informed (verbal and written) consent was obtained from parents/primary caregivers prior to collect data. More details about survey methods can be found elsewhere [33].

In this research, both parent-reported data and adolescent-reported data were merged, and the analyses were restricted to adolescents aged 12-17-years (n = 2521) to preserve age-comparability across the survey and achieve the study objectives. Also, it is done because data on health-risk behaviours (self-harm and suicidality–suicidal ideation, plan and attempt) were only available in self-reported adolescents-data and were strictly limited to 12-17-year-olds age-group.

### Ethics

The Human Research Ethics Committees (HREC) of the UWA (RA/4/1/9197) and AGDH (Project 17/2012) ethically approved the YMM survey [24, 33]. The YMM survey datasets are available on special request at the Australian Data Archive (ADA) Dataverse repository as the YMM datasets contain personal identifying and potentially sensitive information (e.g., mental health, health risk behaviours and service use) about children and adolescents [34]. Hence, following ADA recommended steps in accessing data (https://ada.edu.au/accessing-data/), our research team obtained YMM data access approval from ADA in 2016. In addition, the authorship team obtained ethical approval from the HREC of the University of Southern Queensland (USQ) in 2016 (HREC Approval No. H16REA205) to conduct research using YMM datasets.

### Measures

#### Bullying victimization

In the YMM study, adolescents were directly questioned whether they experienced traditional bullying and/or cyberbullying in the past twelve months. The Revised Olweus Bully/Victim Questionnaire (OBVQ) and the questionnaire from the Cyber Friendly School Project, Edith Cowan University were used to incorporate the items measuring bullying victimization [35–37]. Included questions were as follows: *‘In the past 12 months*, *have you ever been bullied or cyberbullied*?*’ with the listed bullying types–‘Hit*, *kicked*, *or pushed around’*, *‘Made fun of or teased in a mean and hurtful way’*, *‘Lies*, *rumours or nasty stories were spread’*, *‘Threatened or made afraid’*, *‘Deliberately ignored*, *left out on purpose or not allowed to join in’*, *‘Other young people stole things or from me*, *or broke or damaged my things deliberately’*, *‘Teased about my race*, *the colour of my skin or my religion’*,*’ Sent nasty messages by email*, *mobile phone*, *or on the internet’*, *‘Nasty messages or pictures were sent about me to other young people via mobile phone*, *internet or email’*, *and ‘Nasty comments or pictures were sent or posted about me on websites (e*.*g*. *Facebook or Twitter)’*. All responses were dichotomous (Yes/No). In the analysis, from all the responses of the questions, a new binary variable was created as ‘bullying victimization’ and coded as 1 (Yes) and 0 (No).

#### Mental disorders

Seven modules of the DISC-IV (Diagnostic Interview Schedule for Children, Version IV) [38, 39] were used to assess the presence of mental disorder in the past 12 months among the study participants. The included mental disorders were major depressive disorder, attention-deficit-hyperactivity-disorder (ADHD), anxiety disorder, and conduct disorder [40, 41]. For the analysis, from the responses of each mental disorder, a new dichotomous variable was created as ‘mental disorder’ with adolescents who diagnosed with any of the four disorders in the past 12 months and coded as 1 (Yes) and 0 (No). Additionally, a new variable was created as ‘number of mental disorders’ from the responses of each mental disorder. Categories were as follows: an adolescent diagnosed with no mental disorder (coded as 0), adolescent diagnosed with single disorder (coded as 1) and an adolescent diagnosed with multiple mental disorders (2 or more) in the previous 12-months (coded as 2).

#### Self-harm and suicidality (suicidal ideation, plan and attempt)

The Standard High School Questionnaire of the Youth Risk Behaviour Surveillance System (YRBSS) [42] were used in the YMM survey to collect information on self-harm and suicidality (suicidal ideation, plan and attempt). In YMM, adolescents aged 12-17-years answered the following question regarding self-harm [27], *“Have you ever deliberately done something to yourself to cause harm or injury*, *without intending to end your own life*?*”*. Like self-harm, suicidality (ideation, plan and attempt) [26] was identified using the following three questions respectively: *“Have you ever seriously consider attempting in the 12 months prior to the interview*?*”*, *“Did you make a plan about how you would attempt suicide in the past 12 months*?*”*, and *“Did you attempt suicide during the previous 12 months”*. Response options for both self-harm and suicidality (ideation, plan and attempt) were coded as 1 (Yes) and 0 (No). Note that regarding self-harm and suicidality, all the information gathered from the adolescents (self-reported data) were kept confidential and not shared with the consenting parents or primary caregivers.

#### Socioeconomic rank variables

In this paper, the equivalized household income (in quintiles) and the Index of Relative Socioeconomic Advantage and Disadvantage (IRSAD) scores (in quintiles) were used as rank variables to quantify income-based and area-based socioeconomic inequalities in adolescent mental health. Equivalised household income is a measure of the economic resources available to each member of a household, which is derived by estimating an equivalence factor on the basis of ‘Modified OECD’ equivalence scale, and then dividing the income by that equivalence factor. Equivalised household income are divided in quintiles, with quintile 1 (Q1, Less than $20,000 per year) for the poorest and quintile 1 (Q5, $67,000 or more per year) for the richest [34].

While the SEIFA (Socio-Economic Indexes for Areas) IRSAD score is an indicator of both relative socio-economic advantage and disadvantage at the area level. IRSAD employs a range of variables of the Census including income, education, occupation, employment and housing characteristics. The SEIFA IRSAD scores are split into quintiles for all areas across Australia, with quintile 1 (Q1, 0–20%) including the lowest 20% of scores for the most disadvantaged areas and quintile 5 (Q5. 80–100%) containing the highest 20% of scores for the most advantaged areas [43].

### Statistical analysis

The analysis of this study is based on the CIs, which is commonly used in health inequalities research. The sign of the CI implies the direction of any correlation between the health variable of interest and socioeconomic status. Its magnitude reflects not only the extent of the association but also the degree of variability of the health component [44, 45]. The value of CI ranges between +1 and -1, with a zero value of CI suggesting no socioeconomic inequality. A negative CI depicts the unequal concentration of the health variable of interest among the poor (pro-poor inequality) and vice-versa (pro-rich inequality). The larger the absolute value of the CIs, the greater the inequalities [9, 46]. However, in the case of a binary or categorical outcome (e.g., whether an adolescent reported bullying victimization or not), the value of the CI depends on the upper and lower limits [47], which can contribute to unreliable comparisons of inequalities as the mean of the health-related variable varies over time and populations [48, 49]. There are two possible ways to tackle this dispute–(a) Wagstaff’s approach to standardize the CIs by dividing with one minus the means of the mental health-related variables [47], and (b) the Erreygers’s correction approach which adjusts the CIs by multiplying it by four times the mean health-related variable [48]. In the analysis, the second approach was used that fulfils all the four properties of rank dependent measures of inequalities [50].

In the analysis, two ranking variables—equivalized household income quintiles and area-based socioeconomic status (IRSAD quintiles) were used to test the robustness of the estimates due to different measures. Sample weights provided in the YMM dataset were applied in descriptive and inequality analyses to account for survey design of the YMM. Stata 14.1 was used for all statistical analyses.

## Results

The sample characteristics of participants included in the analysis are presented in Table 1. In total, cross-sectional data of n = 2521 adolescents were analysed. Nearly 52% of the study population were boys, and more than 60% aged between 15-17-years (Mean = 14.98, SD = 1.72). Most adolescents were Australian (86%) and lived-in cities (64.5%). More than 90% of adolescents were school going and 37.4% of parents completed diploma. Almost 60% of adolescents lived with both biological parents, and a higher proportion of parents were being employed. Concerning socioeconomic status, majority of the adolescents were from middle-higher income families according to both equivalized household income and area-based IRSAD quintiles. Further characteristics (e.g. 95% CI and concentration indices) of the sample are presented in Table 1.

**Table 1:** Sample characteristics (n = 2521).

	n (%)	95% CI^1^	Concentration Index (CI)^2^
Age			0.031
12 to <15	952 (37.8)	0.35–0.39	
≥15 to 17	1569 (62.2)	0.60–0.64	
Gender			0.009
Boys	1301 (51.6)	0.49–0.53	
Girls	1220 (48.4)	0.46–0.50	
Country of Birth			-0.009
Overseas	354 (14.0)	0.12–0.15	
Australia	2167 (86.0)	0.84–0.87	
Place of residence			-0.066***
Cities	1626 (64.5)	0.62–0.66	
Regional	860 (34.1)	0.34–0.35	
Remote	35 (1.4)	0.01–0.02	
Schooling			0.002
No	210 (8.3)	0.07–0.09	
Yes	2311 (91.7)	0.90–0.92	
Parents’ Education			0.259***
Year 10/11	790 (31.3)	0.29–0.33	
Diploma	943 (37.4)	0.35–0.39	
Bachelor	788 (31.3)	0.29–0.33	
Parents’ Employment			0.317***
Unemployed	584 (23.2)	0.21–0.24	
Employed	1937 (76.8)	0.75–0.78	
Family type^3^			-0.401***
Original	1492 (59.2)	0.57–0.61	
Others	1029 (40.8)	0.38–0.42	
Family functioning^4^			-0.046**
Poor	103 (4.1)	0.03–0.05	
Fair	342 (13.6)	0.12–0.14	
Good	652 (25.8)	0.24–0.27	
Very good	1424 (56.5)	0.54–0.58	
Household income quintiles^5^			0.267***
Q1 (Less than $20,000 per year)—Poorest	450 (17.8)	0.16–0.19	
Q2 ($20,000-$32,999)	539 (21.4)	0.19–0.23	
Q3 ($33,000-$44,999)	454 (18.0)	0.16–0.19	
Q4 ($45,000-$66,999)	592 (23.5)	0.21–0.25	
Q5 ($67,000 or more per year)—Richest	486 (19.3)	0.17–0.21	
SEIFA IRSAD quintiles^6^			0.264***
Q1 (0–20%)—Most disadvantaged	388 (15.4)	0.14–0.16	
Q2 (20–40%)	445 (17.7)	0.16–0.19	
Q3 (40–60%)	536 (21.3)	0.19–0.22	
Q4 (60–80%)	555 (22.0)	0.20–0.23	
Q5 (80–100%)—Most advantaged	597 (23.6)	0.22–0.25	

Notes: Data presented in n (%), 95% CI (^1^Confidence Interval)

^2^p-value (*p<0.05, **p<0.01, ***p<0.001)

^3^Family type: Original families means children are natural, adopted, or foster child of both parents, and no step child; other families include step, blended and children from families who are not natural, adopted, foster or step of either parent.

^4^Family functioning: Poor family functioning can be an indicator of mental health problems in children and vice versa. Hence categorized into poor, fair, good and very good.

^5^Equivalised household income quintiles: Equivalised household income is as a measure of the economic resources available to each member of a household. It is derived by calculating an equivalence factor based on ‘Modified OECD’ equivalence scale and then dividing the income by that equivalence factor. Equivalised household income are divided in quintiles, with quintile 1 (Q1, less than $20,000 per year) for the poorest and quintile 1 (Q5, $67,000 or more per year) for the richest.

^6^SEIFA IRSAD quintiles: The SEIFA (Socio-Economic Indexes for Areas) IRSAD (Index of Relative Socio-economic Advantage and Disadvantage) is used to estimate area-level SES. It employs a range of variables of the Census including income, education, occupation, employment, and housing characteristics. Note that the SEIFA IRSAD is a composite index of the economic and social growth of a region in contrast to other areas, and a lowest IRSAD score (Quintile 1, 0–20%) signifies greater disadvantage as well as a lack of advantages in general and highest IRSAD score (Quintile 5, 80–100%) indicates greater advantages as well as a lack of disadvantage at the area level.

- The ‘Don’t know’ responses were omitted.

**Fig 1:**
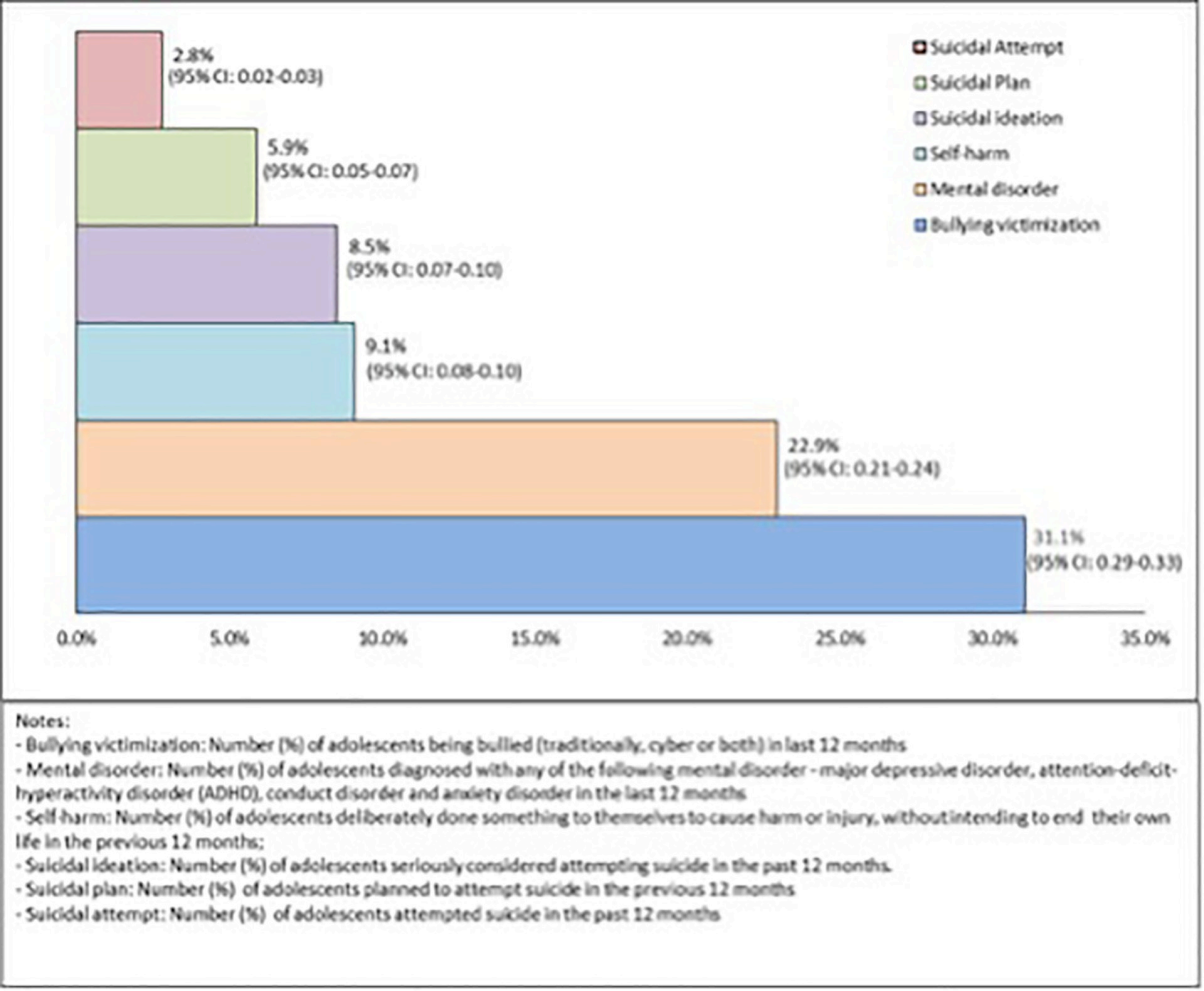
Prevalence (%) of mental health problems with 95% Confidence Interval (CI) in the sample population. Bullying victimization: Number (%) of adolescents being bullied (traditional, cyber or both) in last 12-months. Mental disorder: Number (%) of adolescents diagnosed with any of the following mental disorder—major depressive disorder, attention-deficit-hyperactivity disorder (ADHD), conduct disorder and anxiety disorder in the last 12-months. Self-harm: Number (%) of adolescents deliberately done something to themselves to cause harm or injury, without intending to end their own life in the previous 12-months. Suicidal ideation: Number (%) of adolescents seriously considered attempting suicide in the past 12-months. Suicidal plan: Number (%) of adolescents planned to attempt suicide in the previous 12-months. Suicidal attempt: Number (%) of adolescents attempted suicide in the past 12-months.

Fig 1 shows the prevalence of bullying victimization, mental disorder, self-harm, suicidal ideation, suicidal plan and suicidal attempt among the study participants (n = 2521) were 31.1% (95% CI: 0.29–0.33), 22.9% (95% CI: 0.21–0.24), 9.1% (95% CI: 0.08–0.10), 8.5% (95% CI: 0.07–0.10), 5.9% (95% CI: 0.05–0.07) and 2.8% (95% CI: 0.02–0.03) respectively. The prevalence for mental health problems across equivalized household income quintiles and area-based IRSAD quintiles are presented in Table 2. In the sample, all mental health issues (bullying victimization, number of mental disorders–single and multiple, self-harm, suicidal ideation, plan and attempt) were found to be more prevalent among the poorest and most disadvantaged group (Table 2).

**Table 2:** Prevalence (%) of mental health problems by household income quintiles and area-based IRSAD quintiles.

	Total	Equivalised household income quintiles					Area-based IRSAD quintiles				
		Q1 (Poorest)	Q2	Q3	Q4	Q5 (Richest)	Q1 (Most disadvantaged)	Q2	Q3	Q4	Q5 (Most advantaged)
Bullying victimization											
Yes	784	157 (20.0)	181 (23.1)	132 (16.8)	178 (22.7)	136 (17.4)	130 (16.6)	144 (18.4)	173 (22.1)	173 (22.1)	164 (20.9)
Mental disorder											
Single	411	94 (22.9)	94 (22.9)	65 (15.8)	92 (22.3)	66 (16.1)	75 (18.3)	80 (19.5)	81 (19.7)	96 (23.3)	79 (19.2)
Multiple (2 or more)	167	47 (28.1)	46 (27.5)	27 (16.2)	25 (15.0)	22 (13.2)	39 (23.3)	38 (22.7)	34 (20.4)	27 (16.2)	29 (17.4)
Self-harm											
Yes	229	54 (23.6)	42 (18.3)	42 (18.3)	51 (22.3)	40 (17.5)	43 (18.8)	34 (14.9)	48 (20.9)	47 (20.5)	57 (24.9)
Suicidal ideation											
Yes	213	49 (23.0)	50 (23.5)	35 (16.4)	48 (22.5)	31 (14.6)	46 (21.6)	34 (15.9)	45 (21.1)	39 (18.3)	49 (23.0)
Suicidal plan											
Yes	149	36 (24.2)	31 (20.8)	24 (16.1)	37 (24.8)	31 (14.1)	31 (20.8)	23 (15.4)	30 (20.1)	28 (18.8)	37 (24.8)
Suicidal attempt											
Yes	70	18 (25.7)	18 (25.7)	14 (20.0)	13 (18.6)	7 (10.0)	16 (22.9)	12 (17.1)	16 (22.9)	6 (8.6)	20 (28.6)

Table 3 reports inequality indices for the six outcome variables measured using the Erreygers’s correction. The concentration indices were negative and statistically significant for bullying victimization (CI = -0.049, p = 0.020), multiple mental disorders (CI = -0.088, p = <0.001), suicidal ideation (CI = -0.049, p = 0.047) and suicidal attempt (CI = -0.021, p = 0.002) except for single mental disorder, self-harm and suicidal plan. This indicates that the adolescents from economically worse-off families experienced more mental health issues than those who were from economically better-off, implying a pro-poor inequality in Australia.

**Table 3:** The Erreyger’s CIs for mental health problems among Australian adolescents (12–17 years).

	Equivalised Household Income quintiles			Area-based IRSAD quintiles		
	Concentration Index (CI)	Standard Error of CI	p-value	Concentration Index (CI)	Standard Error of CI	p-value
Bullying victimization	-0.049	0.021	0.020	-0.050	0.021	0.016
Number of mental disorders						
Single	-0.036	0.021	0.090	-0.042	0.021	0.047
Multiple (2 or more)	-0.088	0.013	<0.001	-0.081	0.013	<0.001
Self-harm	-0.017	0.012	0.154	-0.010	0.012	0.379
Suicidal ideation	-0.023	0.011	0.047	-0.024	0.011	0.048
Suicidal plan	-0.013	0.010	0.172	-0.009	0.010	0.333
Suicidal attempt	-0.021	0.006	0.002	-0.011	0.006	0.095

In contrast, there was no notable change revealed in the findings when we used SEIFA IRSAD quintiles instead of household income quintiles in estimating inequality indices in adolescent mental health (Table 3). This signifies that the extent of CIs was almost similar regardless of whether using an equivalized household income quintile or area-based SEIFA IRSAD quintiles.

## Discussion

This research describes the socioeconomic inequality of common mental health issues such as bullying victimization, mental disorder, self-harm, and suicidality (suicidal ideation, plan and attempt) among nationally representative adolescents in Australia, by implementing a concentration index (CI) approach. The current study revealed that, although the magnitude of the socioeconomic inequality was not large, mental health problems were unduly concentrated among adolescents from poor socioeconomic families in Australia. The findings were consistent with the similar studies that indicated a higher prevalence of behavioural/mental disorders in adolescents from low-income households, as well as clear consequences for mental health of adolescents [2, 51]. Pickett and Wilkinson [52] also found a strong relationship between income inequality and mental illnesses across 12 rich countries in the world including Australia. In addition, a cross-national survey involving 31 European countries [53] and a meta-analysis [54] found that mental health problems are common in countries with greater socioeconomic inequalities.

This study substantiated the findings of other studies [6, 55, 56] that the prevalence of bullying victims is disproportionately high among adolescents from low-income families, implying pro-poor socioeconomic inequalities. Moreover, a multilevel study of adolescents in 37 countries confirmed that bullying victimization is significantly associated with income inequality [5]. One mechanism behind this may be the embrace of hierarchies and of having a more divided society that manifested in adolescent’s behaviour [55, 56], as Wilkinson and Pickett [57] explain socioeconomic inequality as a type of structural violence that stimulates disgrace, embarrassment, and violent reprisal.

Similarly, to be consistent with previous research findings [2, 54, 57, 58] the current study found that the burden of personality and multiple mental disorders in an individual was higher among adolescents from lower socioeconomic households compared to their counterparts. This is because the human brain’s dominance behavioural system is more likely to be involved in a wide array of behavioural and mental health problems as they process questions of social superiority and subordination [59]. In particular, the researchers advised that externalizing disorders such as ADHD and conduct disorder are linked to increased desire for superiority, whereas depressive and anxiety disorders are correlated with subordination and obedience [52, 60].

Moreover, the findings of the study show suicidal ideation and suicidal attempt were unequally concentrated among adolescents from economically worse-off families in Australia. This was consistent across different countries, age, gender, and different indexes such as household-income and/or area-level SES [58, 61]. While the study found that self-harming behaviour and suicidal plan were particularly concentrated among adolescents from poor-income families, but not statistically significant. Though previous research reported that low parental socioeconomic conditions are significantly associated with self-harm among adolescents [10, 62]. The increased risk of suicidality and self-harming behaviour attributed to low SES can be supported by a few mechanisms. First, adolescents in adverse conditions in socially disadvantaged households are may be vulnerable to many stressors and are more prone to mental health problems [63]. Second, low socioeconomic condition may be linked with a wide range of undesirable parental consequences such as substance misuse, unemployment, poor family functioning due to divorce or parental separation, mental and/or physical disorders [12, 64], which could affect parenting [65]. A third underlying cause may be social isolation, which can result in decreased self-esteem, feelings of solitude, and depressive symptoms including suicidal ideation and self-harm behaviours during adolescence [62, 66].

Given the strengths of this study, few limitations need to be considered. First, information on bullying, self-harm and suicidality (suicidal ideation, plan and attempt) was from self-reported adolescent-data, which was not validated by any screening tools; may be resulting in overestimation. Second, recall bias may be a concern as mental disorders in adolescents were mostly gathered by parent-data. Third, indicators of socioeconomic rank were measured only by parent-reports, which may include social desirability bias. Lastly, since the data comes from a cross-sectional analysis, causality is difficult to identify. Lastly, some argue that for future research, there needs to be better clarity surrounding the definition of terms used to measure the prevalence of bullying victimization among various age-groups [67] to ensure that conceptual constructs are measured with consistency to ensure the appropriate reporting of bullying victimization especially among children and adolescents.

Yet, the implications of this empirical findings are relatively straight forward. If adolescents are experiencing mental health problems such as bullying victimization, depression, anxiety, self-harming and suicidal behaviours because of low social status, shame, and stigma, they must be handled with dignity and respect for their human worth [52]. In addition, as researchers suggested policy interventions should target to redistribute wealth through taxation and benefits, find ways to reduce sector income gaps before taxes [44, 52], or both to make developed countries like Australia a prosperous and healthier country. Moreover, the findings of the study suggest that more research on the changes in mental health inequality and sociodemographic factors affecting inequalities over time are required to better understand the underlying causes and current distribution of mental health problems among adolescents in Australia. With better detection and reporting of adolescent mental health issues, better prevention and intervention measures can then be developed within the health system and community [68].

## Conclusion

Adolescents from families with lower income in Australia are at higher risk of suffering from different mental health problems including bullying victimization, mental disorder, suicidality (ideation and attempt). This clear evidence of disparity warrants the need to establish tailored intervention approaches to tackle the rising issue of behavioural and mental health problems among adolescents in developed countries like Australia.
